# Reliable estimation of SARS-CoV-2 anti-spike protein IgG titers from single dilution optical density values in serologic surveys

**DOI:** 10.1016/j.diagmicrobio.2022.115807

**Published:** 2022-12

**Authors:** Emilia M.M. Andrade Belitardo, Nivison Nery, Juan P. Aguilar Ticona, Moyra Machado Portilho, Iago O. Mello, Guilherme S. Ribeiro, Mitermayer G. Reis, Federico Costa, Derek A.T. Cummings, Albert I. Ko, Mariam O. Fofana

**Affiliations:** a Instituto Gonçalo Moniz, Fundação Oswaldo Cruz, Salvador, Bahia, Brazil; b Faculdade de Medicina, Universidade Federal da Bahia, Salvador, Bahia, Brazil; c Instituto de Saúde Coletiva, Universidade Federal da Bahia, Salvador, Bahia, Brazil; d Department of Biology, University of Florida, Gainesville, FL, USA; e Emerging Pathogens Institute, University of Florida, Gainesville, FL, USA; f Department of Epidemiology of Microbial Diseases, Yale School of Public Health, New Haven, CT, USA

**Keywords:** SARS-CoV-2, Serology, Antibody, Optical density, Titers

## Abstract

Reliable and scalable seroepidemiology methods are needed to estimate SARS-CoV-2 incidence and monitor the dynamics of population-level immunity as the pandemic evolves. We aimed to evaluate the reliability of SARS-CoV-2 normalized ELISA optical density (nOD) at a single dilution compared to titers derived from serial dilutions. We conducted serial serosurveys within a community-based cohort in Salvador, Brazil. Anti-S IgG ELISA (Euroimmun AG) was performed with 5 serial 3-fold dilutions of paired sera from 54 participants. Changes in nOD reliably predicted increases and decreases in titers (98.1% agreement, κ = 95.8%). Fitting the relationship between nOD and interpolated titers to a log-log curve yields highly accurate predictions of titers (r^2^ = 0.995) and changes in titers (r^2^ = 0.975), using only 1 to 2 dilutions. This approach can significantly reduce the time, labor and resources needed for large-scale serosurveys to ascertain population-level changes in exposure and immunity.

## Highlights

• Single-dilution ELISA optical density reliably predicts SARS-CoV-2 anti-S IgG titers.
• Addition of a second dilution further improves prediction accuracy.
• Changes in predicted titers are highly predictive of changes in measured titers.
• Using predicted titers greatly reduces the resource needs for large-scale serosurveys.

## 1 Introduction

Previous studies have shown that the immune response to SARS-CoV-2 infection results in the development of multiple immunoglobulin classes (IgM, IgA and IgG) as early as the first week after the onset of symptoms [1,2]. Serological assays are essential for epidemiological surveillance and to further the scientific understanding of SARS-CoV-2 immunity by monitoring the dynamics of population-level immunity as infections, vaccination and waning occur, and the resulting impact on transmission [3], [4], [5], [6].

Whereas the qualitative presence or absence of antibodies provides meaningful information in non-immune individuals, in populations that have been highly exposed to infection and vaccination, ascertaining new infections requires assessing quantitative changes in antibody levels. The determination of binding antibody titers is typically very labor- and resource-intensive, as it requires measuring the presence of antibodies above a given threshold at multiple serial dilutions. Reducing the time and effort necessary for quantitation of antibody levels can help to expedite studies of immune response among individuals with exposure to SARS-CoV-2 vaccination or infection. Simpler and less costly methods of quantitation would be particularly valuable in resource-limited settings where laboratory capacity, staff, materials and reagents are scarce.

We therefore sought to assess whether the normalized ELISA optical density (OD) values at a single dilution could accurately estimate titers derived from serial dilutions, and changes in titers over time.

## 2 Materials and methods

### 2.1 Study site and population

**Fig. 1:**
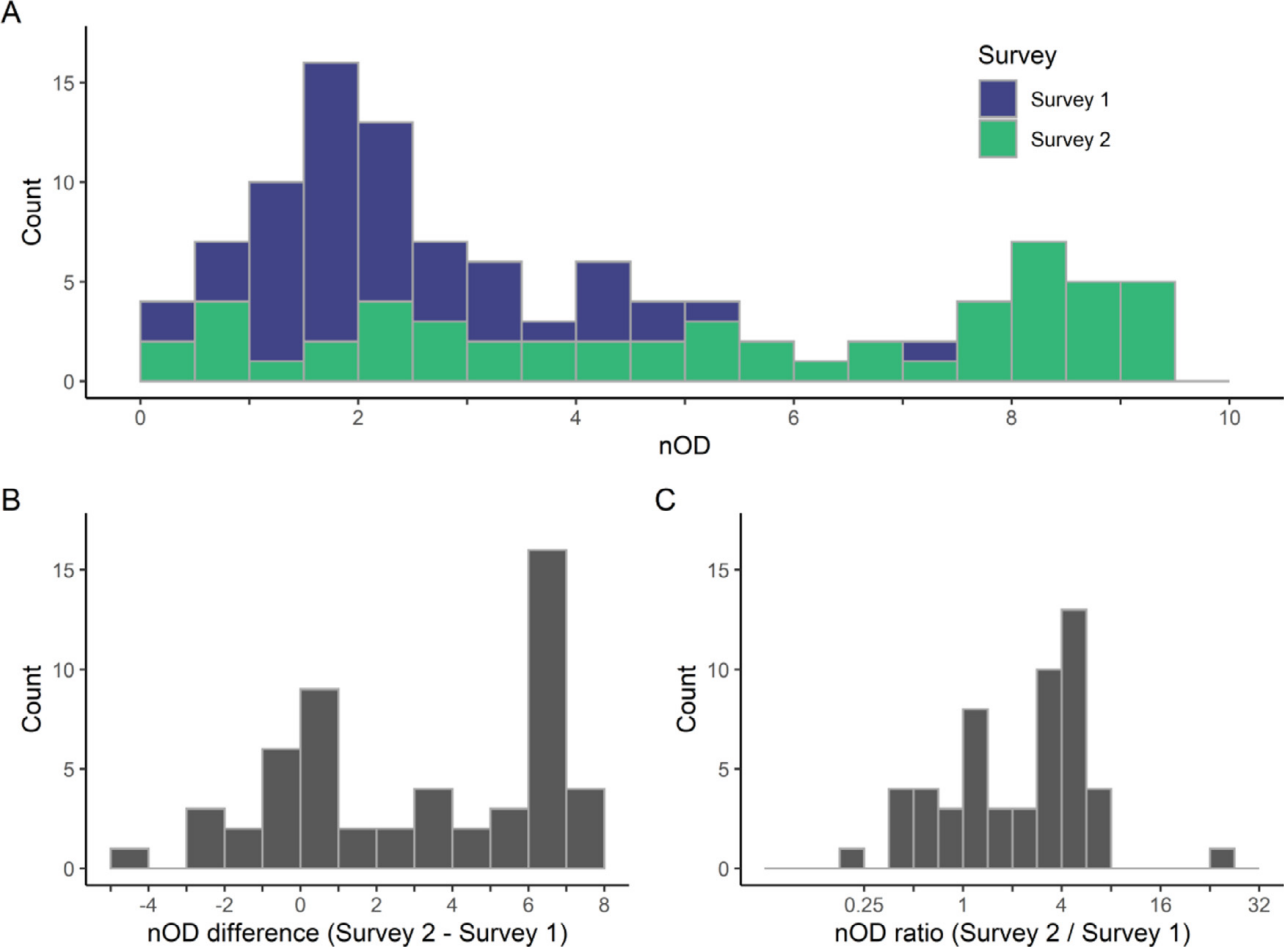
Distribution of samples selected for titer measurements. (A) nOD values (1:101 dilution) of samples collected during Survey 1 (blue) and Survey 2 (green). (B) Difference in nOD values between Survey 2 and Survey 1. (C) Ratio of nOD values (Survey 2: Survey 1).

This study was conducted within an open cohort of residents in the Pau da Lima community, located in Salvador, Brazil. Household-based serological surveys have been conducted regularly at this site for several years to study emerging infections [7], [8], [9], [10]. Individuals who sleep 3 or more nights per week within the defined study area, are aged 2 years or older, and who provide consent (parental consent for minors) were eligible to participate. Serological samples were collected from November 18, 2020 to February 26, 2021, after the first COVID-19 epidemic wave, and from July 14, 2021 to October 31, 2021, after the second wave, to evaluate seroprevalence and longitudinal trends in antibody response. A total of 1,571 individuals had paired longitudinal samples from both surveys. For the primary analysis we selected a sample of 54 individuals, aiming to achieve representation of a broad range of normalized OD (nOD) and titer values to fully characterize the relationship between these measurements (Fig. 1). This sample included 49 individuals who were seropositive during the first survey, of whom 18 had received at least 1 vaccine dose prior to the second survey. The remaining 5 individuals had no evidence of SARS-CoV-2 infection and had not received a vaccine. The ranges of nOD values (1:101 dilution) and changes in nOD values among this sample are shown in Fig. 1.

In order to validate our prediction model, we selected another 40 individuals with paired samples from both surveys, including 13 individuals with evidence of infection before the first survey, 13 individuals with no evidence of infection and who received at least 1 vaccine dose between the first and second surveys, and 14 individuals with evidence of infection before the first survey who also received at least 1 vaccine dose between the first and second surveys. Additionally, 333 banked samples collected from cohort participants between September 9 and November 11, 2019, prior to the emergence of COVID-19 in Brazil, served as negative controls. These samples were not used for the development or validation of the prediction model.

### 2.2 Ethical and confidentiality considerations

Study participants were informed about the project, the risks and the absence of immediate individual benefits. Participation in the study was voluntary and could be interrupted at any time. All adult participants signed an informed consent form in the presence of witnesses prior to enrollment, in accordance with Resolution no. 466/2012 of the Brazil Ministry of Health. Parental consent was obtained for minors. This project was approved by the Human Research Ethics Committee of the Instituto Gonçalo Moniz, Fundação Oswaldo Cruz (FIOCRUZ), the National Research Ethics Council (CONEP) and the Yale University Research Ethics Committee.

### 2.3 Collection, transport and storage of samples

Trained members of the study team performed venipuncture. Blood samples were collected in a dry tube and transported to FIOCRUZ in refrigerated transport boxes. After centrifugation at 3000 RPM (1,811 RCF) at 4°C for 15 minutes, the samples were aliquoted and stored at -20°C.

### 2.4 SARS-CoV-2 IgG ELISA and titers

Serological assays for detection of IgG against the SARS-CoV-2 spike protein were performed using commercial ELISA kits (Euroimmun AG, Lübeck, Germany) and plates pre-coated with the S1 domain of wild-type (Wuhan-Hu-1) SARS-CoV-2, expressed recombinantly in the human cell line HEK 293. Samples were diluted 1:101 in buffer and processed according to the manufacturer's instructions. Briefly, 100µl of each sample, a calibrator, and positive and negative controls were added to the plate and incubated for 1 hour at 37°C. After 3 wash steps with wash buffer, 100µl of HRP-labeled secondary anti-human IgG was added for 30 minutes at 37°C. The plates were washed 3 more times with wash buffer and 100µl of substrate solution (TMB/H_2_O_2_) was added for 30 minutes at room temperature, with shielding from light. The reaction was stopped with the addition of 100µl of 0.5M sulfuric acid and the absorbance was measured at a wavelength of 450nm using an automated plate reader (Tecan Austria GmbH, Grӧdig, Austria). Normalized OD values were calculated as the ratio of the OD of each test sample to that of the calibrator.

Titers were obtained by qualitative assessment of antibody binding at 5 serial 3-fold dilutions (1:100, 1:300, 1:900, 1:2700, 1:8100), based on previously published protocols [11]. Interpolated titers were computed using the software GraphPad Prism (Version 5.01, GraphPad software, San Diego, USA). These values correspond to the estimated titer at which the presence of antibody is no longer detected, interpolated from the highest dilution with positive antibody detection and the next serial dilution. Unlike endpoint titers, which are interval-censored, interpolated titers are on a continuous scale, and result in less biased estimates [12]. All assays in the primary analysis (*N* = 54) were conducted by a single operator, on 35 distinct days. Assays for the validation analysis (*N* = 40) were performed by the same operator. ELISA nOD and serial titer measurements were repeated by a different operator to assess precision across 5 replicates in a subset of samples.

### 2.5 Threshold for antibody detection

For the Euroimmun anti-S IgG ELISA, the manufacturer recommends that samples with normalized OD values < 0.8 are considered negative, those with values ≥ 0.8 and < 1.1 considered borderline, and those with values ≥ 1.1 considered positive. We defined samples with values ≥ 0.8 as positive. In order to evaluate the appropriateness of these cutoffs, which were derived from evaluation of COVID-19 patients, to the context of a seroprevalence survey in our study population, we performed assays using pre-pandemic serum samples as negative controls. We then applied a widely accepted method to establish cutoffs in the absence of known positive standards, using the upper prediction limit from negative samples [13]. Using this method, at a desired significance level of 99.9%, the derived cutoff is 0.81, similar to the lower manufacturer-suggested cutoff (Table S1).

### 2.6 Statistical analysis

We compared interpolated titers to the nOD values obtained at the 1:100 dilution and at the 1:8100 dilution among the 54 individuals (108 samples) selected for the primary analysis. The relationships between these values fit sigmoidal functions, consistent with previous observations [14]. We fit 5-parameter log-log curves to the data using the R packages “aomisc” and “drc” [15,16]. We assessed both the Pearson and Spearman (rank) correlation coefficients for the change in nOD and change in interpolated titers. Given that a 4-fold change in titers is a common criterion to identify recent antigen exposure such as would occur with incident infection [17,18], we computed the area under the ROC curve for the change in nOD values that would correspond to 4-fold increases or decreases in interpolated titers.

Using the parametric fits from the primary analyses, we derived predicted titers among the 40 individuals (80 samples) selected for the validation analysis. We derived predicted titers from (1) the fit for nOD at a 1:100 dilution, (2) the fit for nOD at a 1:8100 dilution, or (3) a stepwise approach combining the fits for nOD at a 1:100 dilution and a 1:8100 dilution. We then compared the predicted titers to measured titers from serial dilutions. After imputing predicted titer values of 0 for samples with nOD below the manufacturer-reported limit of detection (0.078) at the lowest (1:100) dilution, we computed the change in predicted titers and compared it to the change in measured titers between the 2 surveys. Accuracy was assessed using the Pearson and Spearman correlation coefficients. We repeated measurements of nOD and serial-dilution titers in a subset of 5 samples from the validation set, and we computed the coefficient of variation across 5 replicates to assess precision. All analyses were conducted using the software R, version 4.1.3 [19]. Analysis datasets and code are available at: https://github.com/m-fofana/SARS-CoV-2-Serology.

## 3 Results

### 3.1 Estimation of interpolated titers using normalized OD

**Fig. 2:**
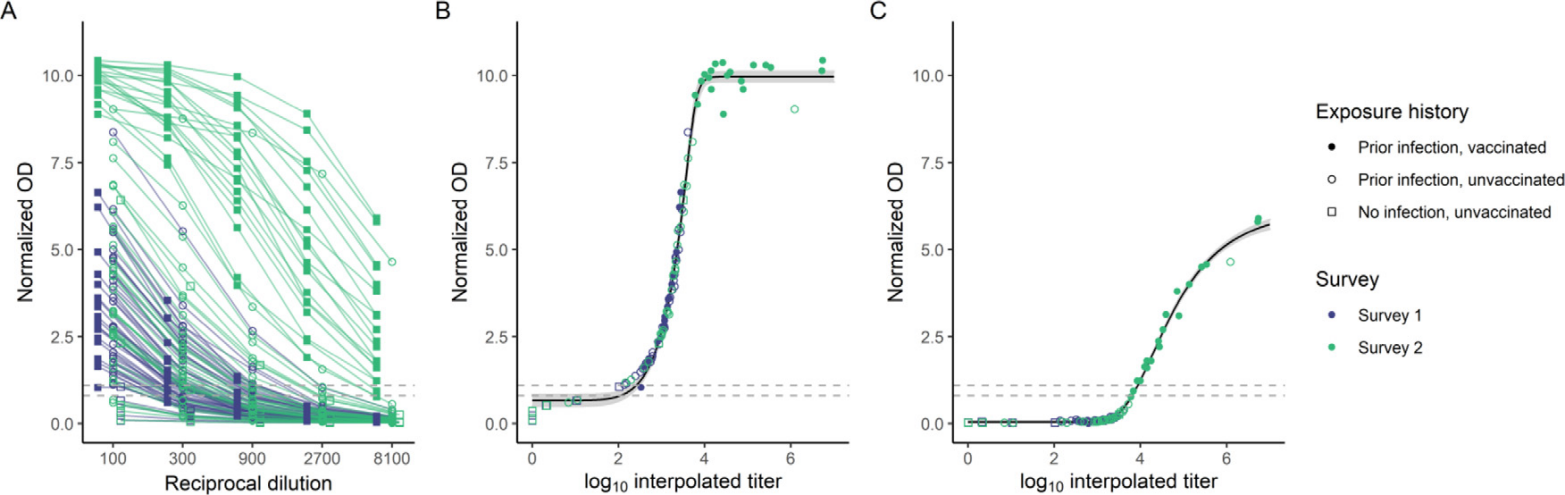
Anti-S IgG measurements. (A) Summary of normalized OD values obtained at each serial dilution. Each curve represents a single sample collected during Survey 1 (blue) or Survey 2 (green). (B) Comparison of the interpolated titer as estimated from serial dilutions to the nOD measured at a single dilution of 1:100 revealed a sigmoidal relationship. The solid black line shows fitted values based on a 5-parameter log-log curve, with the 95% confidence interval shown in gray. (C) Comparison of the interpolated titer as estimated from serial dilutions to the nOD measured at a single dilution of 1:8100 discriminates better between high titer values. Dashed gray lines in A-C represent the manufacturer-suggested nOD cutoffs of 0.8 and 1.1. Solid circles represent individuals with evidence of SARS-CoV-2 infection during Survey 1 and who subsequently received at least one vaccine dose prior to Survey 2. Hollow circles represent individuals with prior infection who did not receive vaccination prior to Survey 2. Hollow squares represent individuals who had no evidence of infection during Survey 1 and did not receive vaccination prior to Survey 2.

Fig. 2A shows the nOD values at each of 5 3-fold serial dilutions for the 108 paired samples from Survey 1 (blue) and Survey 2 (green) included in our primary analysis, stratified by prior exposure to infection and/or vaccination. We estimated interpolated titers using a cutoff of nOD ≥ 0.8 for positive antibody detection. The relationship between interpolated titers and nOD at a 1:100 dilution exhibited a clear sigmoidal curve pattern (Fig. 2B). We fitted the data to a 5-parameter log-log curve, with the following form: $Y=c+\frac{d-c}{{\left(1+{\left(\frac{x}{e}\right)}^{b}\right)}^{f}}$ where *c* and *d* are the lower and upper asymptotes, respectively, and *f* is an asymmetry parameter. Parameters *b* and *e* characterize the Hill's slope and inflection point. We similarly fitted 5-parameter log-log curves for the relationship between nOD at a 1:8100 dilution (Fig. 2C). Details of the model parameters are shown in Table S2.

### 3.2 Prediction of changes in serostatus

**Fig. 3:**
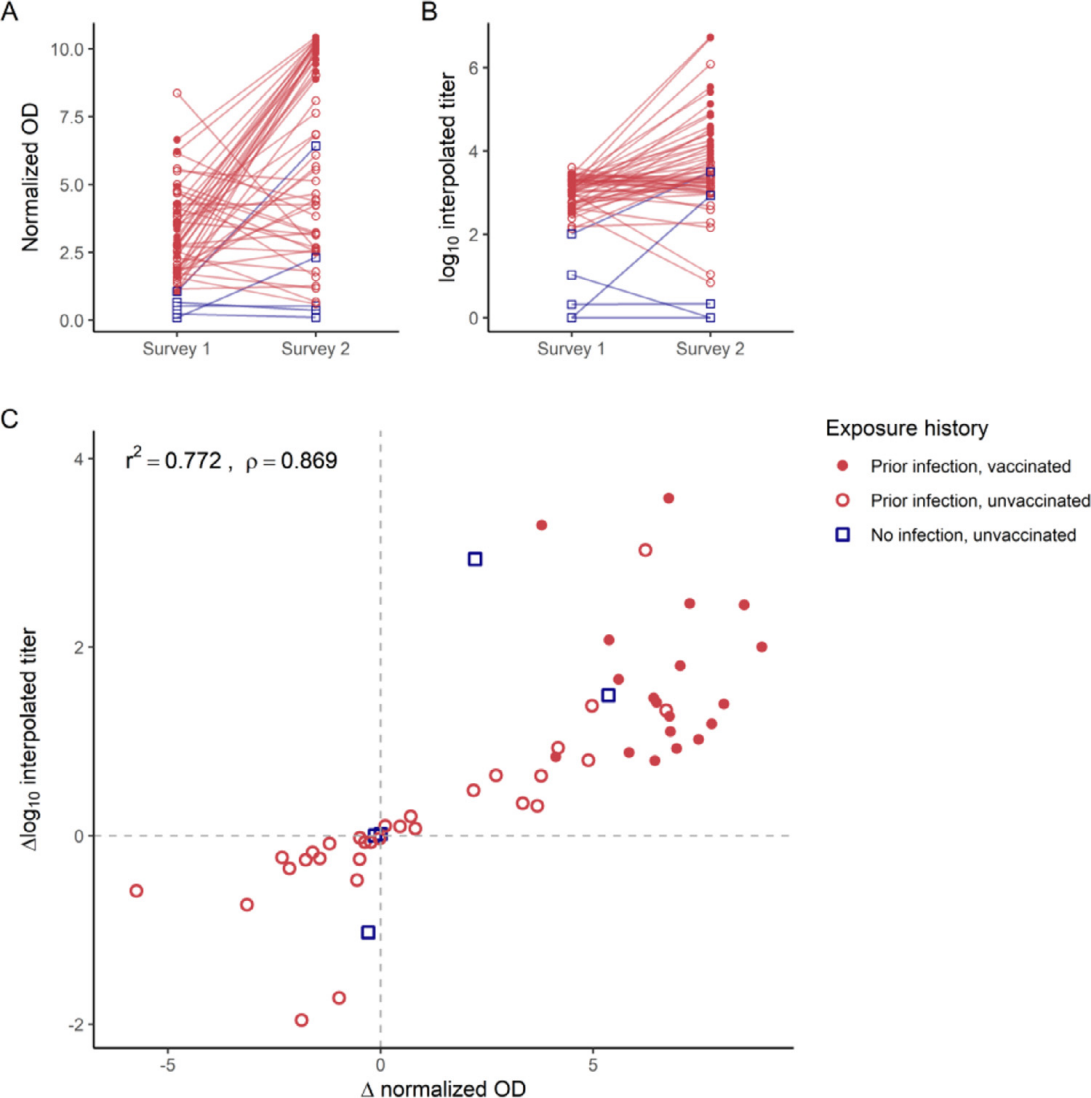
Change in normalized OD and titers. Spaghetti plots of individual change in nOD at a 1:100 dilution (A) and interpolated titer (B) between Survey 1 and Survey 2. Panel C illustrates the concordance in the direction of change (increase vs. increase) and the correlation between the difference in interpolated titer and the difference in nOD at a 1:100 dilution. Each data point represents the difference between Surveys 1 and 2 for the same individual. Dashed gray lines in indicate no change in nOD or titer. A-C: Solid circles represent individuals with evidence of SARS-CoV-2 infection during Survey 1 and who subsequently received at least 1 vaccine dose prior to Survey 2. Hollow circles represent individuals with prior infection who did not receive vaccination prior to Survey 2. Hollow squares represent individuals who had no evidence of infection during Survey 1 and did not receive vaccination prior to Survey 2.

We compared the observed changes in interpolated titers to the changes in nOD at a single 1:100 dilution (Fig. 3). Overall, there was a good correlation between the difference in interpolated titer and the difference in nOD between paired samples from Survey 1 and Survey 2 (Pearson correlation r^2^ = 0.772, Spearman correlation ρ = 0.869; Fig. 3 C). There was 98.1% concordance (κ = 95.8%) for the detection of an increase or decrease in interpolated titer. The area under the receiver operating characteristic curve (AUC) values for the detection of a four-fold increase or decrease in interpolated titer were 99.2% (95% confidence interval [CI] 97.7−100.0%) and 97.5% (95% CI 93.4−100.0%). A 1.5-fold change in nOD predicted a 4-fold rise in interpolated titer with 100.0% sensitivity and 88.5% specificity. A 2.1-fold change in nOD was more specific (100.0%) but less sensitive (92.9%).

### 3.3 Validation of prediction approach

**Fig. 4:**
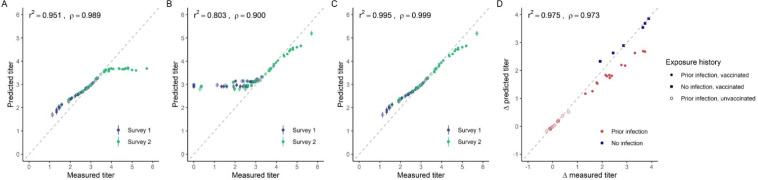
Accuracy of titer prediction from normalized OD. Accuracy of prediction of interpolated titers using nOD at a single dilution of 1:100 (A) or 1:8100 (B). A stepwise approach whereby samples with nOD >7 at the 1:100 dilution undergo nOD measurement and titer prediction at a 1:8100 dilution improves accuracy (C). Changes in titers predicted from nOD at 1 or 2 dilutions (1:100, 1:8100) are highly correlated with changes in titers derived from serial dilutions (D). A-D: the gray dashed line indicates identity (y = x). Solid circles represent individuals with evidence of SARS-CoV-2 infection during Survey 1 and who subsequently received at least 1 vaccine dose prior to Survey 2. Solid squares represent individuals who had no evidence of infection during Survey 1 and received at least 1 vaccine dose prior to Survey 2. Hollow circles represent individuals with prior infection who did not receive vaccination prior to Survey 2. The vertical lines overlying each point represent the 95% confidence interval. Colors represent the time of sampling (A-C) or prior infection status (D).

Using the model fits from the primary analysis, we derived predicted titers in a distinct set of 40 individuals with paired samples from Surveys 1 and 2, using nOD values at a 1:100 or 1:8100 dilution. We compared the titers predicted from a single nOD value to the measured titers derived from serial dilutions. Predictions using the 1:100 dilution nOD performed better at the lower end of the range of values (Fig. 4A), whereas predictions using the 1:8100 dilution nOD performed better at the upper end of the range (Fig. 4B). We therefore implemented a stepwise approach, whereby we derived an initial prediction of titer from the nOD value at the 1:100 dilution. For samples with nOD > 7 at the 1:100 dilution, predictions were derived from the nOD value at the 1:8100 dilution, yielding highly accurate predictions (Pearson correlation r^2^ = 0.995, Spearman correlation ρ = 0.999; Fig. 4C). This approach would have reduced the total number of dilutions required for the 188 samples included in this study 4.14-fold, from 940 (5 dilutions per sample) to 227 (1 dilution for the 149 samples with 1:100 nOD ≤ 7 and 2 dilutions for the 39 samples with 1:100 nOD >7). The estimated interpolated titers corresponding to a wide range of nOD values (1:100 and/or 1:8100 dilution) are shown in Table S3.

### 3.4 Precision and sensitivity analyses

Table 1 shows the range, mean, standard deviation, and coefficient of variation for 5 replicates of nOD and titer values. Variability across single-dilution nOD measurements was acceptable, with a coefficient of variation ranging from 1.75% to 6.41% (manufacturer-reported range 2.4%−9.0%) [20]. Titers showed less variability across measurements overall. With the exception of 1 sample with values below the threshold of positivity even at the lowest (1:100 dilution), the coefficient of variation for interpolated titers ranged from 0.19% to 3.03%. We repeated our primary analyses using a cutoff of 1.1 for presence of anti-spike protein antibodies and did not observe any significant changes to our findings (Fig. S1).

**Table 1:** Intra-assay precision.

Sample ID	Normalized OD				Interpolated titer			
	Range	Mean	SD	% CV	Range	Mean	SD	% CV
172	0.475−0.550	0.507	0.027	5.39	0.182−0.498	0.311	0.133	42.91
407	1.250−1.465	1.338	0.086	6.41	2.315−2.491	2.383	0.072	3.03
400	2.300−2.625	2.491	0.154	6.17	2.941−3.026	2.987	0.037	1.24
274	3.225−3.558	3.377	0.124	3.68	3.141−3.213	3.170	0.027	0.84
242	6.791−7.075	6.948	0.122	1.75	3.639−3.657	3.647	0.007	0.19

## 4 Discussion

### 4.1 Key findings

We found a high correlation between ELISA nOD values at a single 1:100 dilution and interpolated titers derived from serial dilutions. We were able to fit the relationship between normalized OD and interpolated titers to a parametric log-log curve, such that nOD values could be used to estimate corresponding interpolated titers. Our results demonstrate that a single ELISA optical density measurement using a widely available commercial assay can reliably estimate SARS-CoV-2 IgG titers for population-level serological surveys. Prediction accuracy is further improved by a stepwise approach, with addition of a second (1:8100) dilution for samples with high nOD values (nOD >7 at 1:100 dilution). Moreover, we found a high correlation between changes in predicted titers and changes in measured titers between paired serial samples from the same individuals. Applying this prediction approach would have reduced the number of sample runs required in this study by more than 4-fold.

### 4.2 Limitations

One limitation of this work is that we did not assess the correlation between nOD values and virus neutralization activity. Nevertheless, prior studies have demonstrated that binding antibody levels correlate well with neutralizing activity [21]. Although assessment of neutralizing activity is necessary in certain contexts, binding antibody levels are more practical and scalable for applications such as population serological surveys, or assessment of immune response to vaccination. The assays in our study were performed in a single lab and by a 2 operators. There may be additional variability across operators and laboratories. Reassuringly, despite using samples that were collected 6 months apart and processed over several weeks, there was no significant systematic variation. Precision for both single nOD measurements and serial dilution titers was acceptable and similar to the manufacturer-reported range. Although detected antibody response may vary across SARS-CoV-2 variants [22] due to intrinsic factors (e.g., disease severity, immunogenicity) and measurement-related factors (e.g., differential binding to assay antigens due to mutations in the Spike protein), we do not expect that the relationship between single-dilution nOD and serial-dilution titers would differ across variants, as long as both values are derived from the same assays, using the same antigens.

### 4.3 Implications and future directions

There is a continuing need to expand access to SARS-CoV-2 research capability in low- and middle-income countries. Serological surveys are especially important in settings where there may be a lower proportion of symptomatic infections (i.e., younger populations), as they can identify infections that would otherwise go undetected. Our findings raise the possibility that future studies of antibody waning and response to vaccine booster doses could rely on ELISA nOD measurement of paired samples at 1 or 2 dilutions rather than requiring serial titration of each sample, thus greatly reducing the necessary effort and expense. Moreover, our study population included individuals with a broad range of binding antibody levels, allowing us to characterize a broad range of nOD and titer values. Future studies should assess the validity of our prediction approach in other settings, to validate its applicability.

With the continuing rollout of vaccination and boosters, serological surveys will play an important role in research investigating the dynamics of population-level immunity and the resulting impact on transmission. Serial measurements, or combined measurement of IgG and other immunoglobulins, could serve to reconstruct the infection history and immunity dynamics of populations to guide policies on mitigation measures and vaccination (e.g., number of doses, timing, priority populations). Our findings demonstrate that such studies can be conducted with 1-2 ELISA nOD measurements, thus reducing the effort, time, and cost involved. We expect that these gains will be particularly valuable in resource-limited settings where laboratory capacity is strained.

## Funding

This work was supported by grants from the National Institutes of Health (R01 AI052473, U01AI088752, R01 TW009504 and R25 TW009338 to A.I.K.), UK Medical Research Council (MR/T029781/1 to F.C.), Wellcome Trust (102330/Z/13/Z; 218987/Z/19/Z to F.C.), Brazilian Coordination for the Improvement of Higher Education Personnel (CAPES88881.130749/2016-01 to G.S.R.), the Brazilian National Council for Scientific and Technological Development (CNPq 405308/2021-3 and research scholarship to G.S.R.), the Bahia Foundation for Research Support (FAPESB SUS0019/2021 and PET0022/2016 to G.S.R.), the Bill & Melinda Gates Foundation (OPP1211988 to M.G.R. and F.C.), the American Society of Tropical Medicine and Hygiene (Burroughs Wellcome Fund Postdoctoral Fellowship to M.O.F.) and the Sendas Family Fund at the Yale School of Public Health (A.I.K.). The funders had no role in study design, data collection and analysis, decision to publish, or preparation of the manuscript.

## Declaration of competing interest

A.I.K serves as an expert panel member for Reckitt Global Hygiene Institute, scientific advisory board member for Revelar Biotherapeutics and a consultant for Tata Medical and Diagnostics and Regeneron Pharmaceuticals, and has received grants from Merck, Regeneron Pharmaceuticals and Tata Medical and Diagnostics for research related to COVID-19, all of which are outside the scope of the submitted work. M.O.F has previously held stock in GlaxoSmithKline. Other authors declare no conflict of interest.

## Ethics statement

The study was approved by the Institutional Review Boards of the Gonçalo Moniz Institute, Oswaldo Cruz Foundation (Fiocruz) and the Brazilian National Commission for Ethics in Research (CAAE 35405320.0.1001.5030 and 17963519.0.0000.0040), and the Yale University Human Research Protection Program (2000031554).

## Authors’ contributions

Juan P. Aguilar Ticona: Investigation, Data curation, Writing-review and editing; Emilia M. M. Andrade Belitardo: Conceptualization, Investigation, Writing-original draft; Federico Costa: Supervision, Funding, Writing-review and editing; Derek A. T. Cummings: Conceptualization, Supervision, Writing-review and editing; Mariam O. Fofana: Conceptualization, Formal analysis, Writing-original draft; Albert I. Ko: Conceptualization, Supervision, Funding, Writing-review and editing; Iago O. Mello: Investigation, Writing-review and editing; Nivison Nery Jr: Investigation, Data curation, Writing-review and editing; Moyra M. Portilho: Investigation, Writing-review and editing; Mitermayer G. Reis: Supervision, Writing-review and editing; Guilherme S. Ribeiro: Supervision, Funding, Writing-review and editing.

## Appendix Supplementary materials

Image, application 1
