# Supplementary material for: Reliable estimation of SARS-CoV-2 anti-spike protein IgG titers from single dilution optical density values in serologic surveys
Source: Diagn Microbiol Infect Dis. 2022 Dec;104(4):115807. doi: 10.1016/j.diagmicrobio.2022.115807 (PMC9428330; doi:10.1016/j.diagmicrobio.2022.115807)
Supplement: Supplementary file 1 [file mmc1.docx]

Reliable estimation of SARS-CoV-2 anti-spike protein IgG titers from single dilution optical density values in serologic surveys

Supplementary figures and tables

Emilia M. M. Andrade Belitardo^1^, Nivison Nery Jr^1^, Juan P. Aguilar Ticona^1^, Moyra Machado Portilho^1^, Iago O. Mello^1^, Guilherme S. Ribeiro^1,2^, Mitermayer G. Reis^1,2,6^, Federico Costa^1,3,6^, Derek A. T. Cummings^4,5^, Albert I. Ko^1,6^, Mariam O. Fofana^6*^

1. Instituto Gonçalo Moniz, Fundação Oswaldo Cruz, Salvador BA, Brazil
2. Faculdade de Medicina, Universidade Federal da Bahia, Salvador BA, Brazil
3. Instituto de Saúde Coletiva, Universidade Federal da Bahia, Salvador BA, Brazil
4. Department of Biology, University of Florida, Gainesville FL, USA
5. Emerging Pathogens Institute, University of Florida, Gainesville FL, USA
6. Department of Epidemiology of Microbial Diseases, Yale School of Public Health, New Haven CT, USA

**
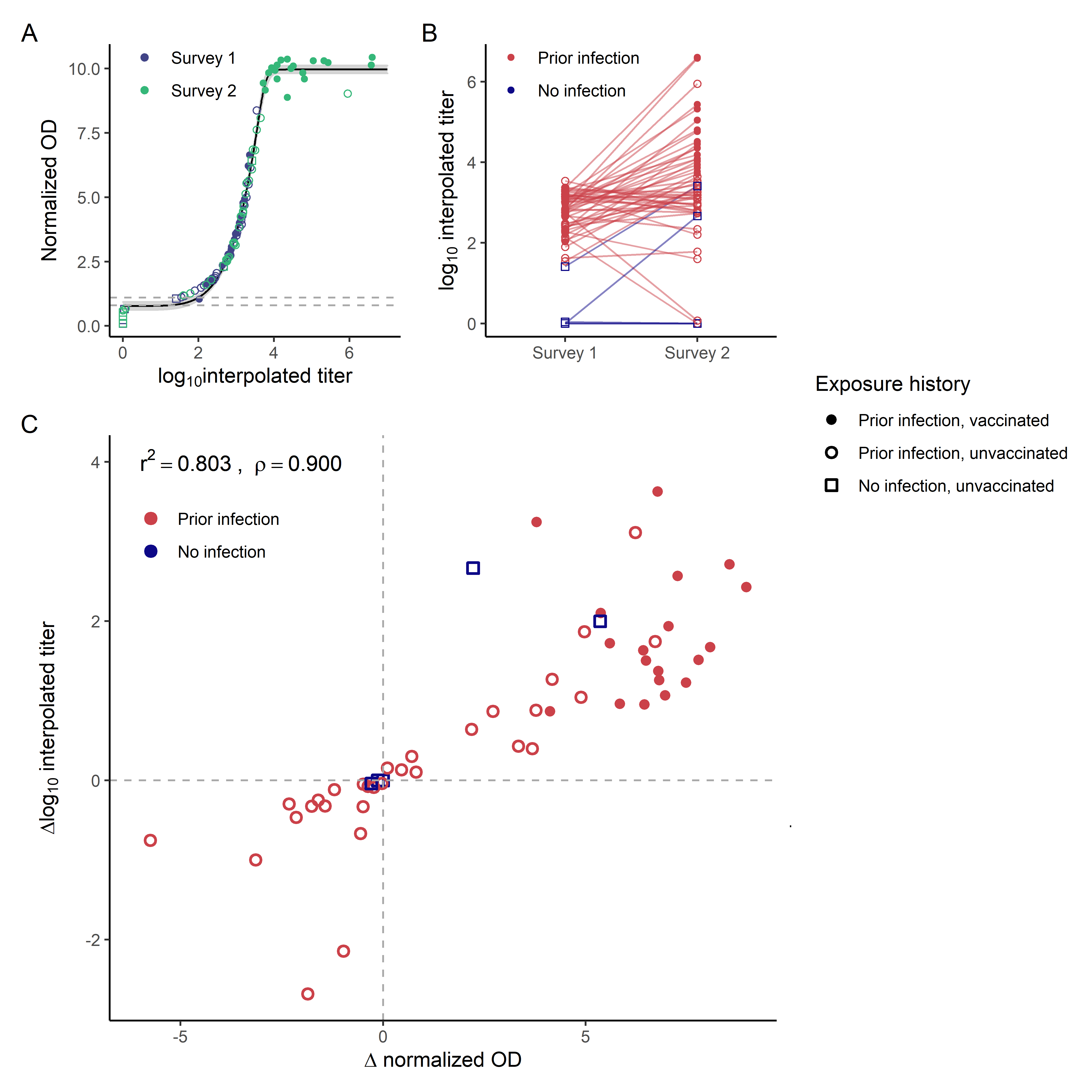
Figure S1: Sensitivity analyses.** We repeated our primary analyses using an nOD cutoff for the presence of anti-S antibody of 1.1 (vs. 0.8). The relationship and parametric fit of nOD values (1:100 dilution) to interpolated titers (A), as well as the correlation between the change in nOD and the change in interpolated titer (B & C) remained similar (r^2^ = 0.836 vs. 0.780, ρ = 0.873 vs. 0.868). A-C: Solid circles represent individuals with evidence of SARS-CoV-2 infection during Survey 1 and who subsequently received at least one vaccine dose prior to Survey 2. Hollow circles represent individuals with prior infection who did not receive vaccination prior to Survey 2. Hollow squares represent individuals who had no evidence of infection during Survey 1 and did not receive vaccination prior to Survey 2. Colors represent the time of sampling (A) or prior infection status (B-C).

**Table S1: OD cutoff values derived from distribution of negative (pre-pandemic) samples**

| α | Raw OD cutoff | Normalized OD cutoff |
| --- | --- | --- |
| 95.0% | 0.190 | 0.499 |
| 97.5% | 0.215 | 0.565 |
| 99.0% | 0.245 | 0.643 |
| 99.5% | 0.265 | 0.696 |
| 99.9% | 0.307 | 0.805 |

**Table S2: Curve fit parameters**

| Parameter | 1:100 dilution nOD | |  | 1:8100 dilution nOD | |
| --- | --- | --- | --- | --- | --- |
|  | Estimate | Standard error |  | Estimate | Standard error |
| b | -40.93 | 8.03 |  | -5.41 | 0.25 |
| c | 0.66 | 0.09 |  | 0.04 | 0.01 |
| d | 9.96 | 0.08 |  | 6.22 | 0.13 |
| e | 3.75 | 0.02 |  | 2.81 | 0.28 |
| f | 0.17 | 0.04 |  | 11.88 | 5.32 |
| *Residual standard error* | *0.304* |  |  | *0.103* |  |

**Table S3: ELISA nOD to titer conversion**

| nOD (1:100) | Estimated titer (95% CI) |
| --- | --- |
|  |  |
| 0.67 | 1.40 (1.28-1.52) |
| 0.70 | 1.68 (1.56-1.80) |
| 0.80 | 2.04 (1.94-2.14) |
| 0.90 | 2.21 (2.11-2.30) |
| 1.00 | 2.32 (2.24-2.41) |
| 1.10 | 2.41 (2.33-2.50) |
| 1.20 | 2.49 (2.41-2.56) |
| 1.30 | 2.55 (2.47-2.62) |
| 1.40 | 2.60 (2.53-2.67) |
| 1.50 | 2.65 (2.58-2.72) |
| 1.60 | 2.69 (2.63-2.76) |
| 1.70 | 2.73 (2.67-2.80) |
| 1.80 | 2.77 (2.71-2.83) |
| 1.90 | 2.80 (2.75-2.86) |
| 2.00 | 2.84 (2.78-2.89) |
| 2.10 | 2.87 (2.82-2.92) |
| 2.20 | 2.89 (2.85-2.94) |
| 2.30 | 2.92 (2.87-2.97) |
| 2.40 | 2.95 (2.90-2.99) |
| 2.50 | 2.97 (2.93-3.01) |
| 2.60 | 2.99 (2.95-3.03) |
| 2.70 | 3.01 (2.97-3.05) |
| 2.80 | 3.03 (3.00-3.07) |
| 2.90 | 3.05 (3.02-3.09) |
| 3.00 | 3.07 (3.04-3.11) |
| 3.10 | 3.09 (3.06-3.13) |
| 3.20 | 3.11 (3.08-3.14) |
| 3.30 | 3.13 (3.10-3.16) |
| 3.40 | 3.15 (3.12-3.17) |
| 3.50 | 3.16 (3.13-3.19) |
| 3.60 | 3.18 (3.15-3.20) |
| 3.70 | 3.19 (3.17-3.22) |
| 3.80 | 3.21 (3.18-3.23) |
| 3.90 | 3.22 (3.20-3.25) |
| 4.00 | 3.24 (3.21-3.26) |
| 4.10 | 3.25 (3.23-3.27) |
| 4.20 | 3.26 (3.24-3.29) |
| 4.30 | 3.28 (3.26-3.30) |
| 4.40 | 3.29 (3.27-3.31) |
| 4.50 | 3.30 (3.28-3.32) |
| 4.60 | 3.31 (3.30-3.33) |
| 4.70 | 3.33 (3.31-3.35) |
| 4.80 | 3.34 (3.32-3.36) |
| 4.90 | 3.35 (3.33-3.37) |
| 5.00 | 3.36 (3.34-3.38) |
| 5.10 | 3.37 (3.36-3.39) |
| 5.20 | 3.38 (3.37-3.40) |
| 5.30 | 3.39 (3.38-3.41) |
| 5.40 | 3.41 (3.39-3.42) |
| 5.50 | 3.42 (3.40-3.43) |
| 5.60 | 3.43 (3.41-3.44) |
| 5.70 | 3.44 (3.42-3.45) |
| 5.80 | 3.45 (3.43-3.46) |
| 5.90 | 3.46 (3.44-3.47) |
| 6.00 | 3.47 (3.45-3.48) |
| 6.10 | 3.48 (3.46-3.49) |
| 6.20 | 3.49 (3.47-3.50) |
| 6.30 | 3.49 (3.48-3.51) |
| 6.40 | 3.50 (3.49-3.52) |
| 6.50 | 3.51 (3.50-3.53) |
| 6.60 | 3.52 (3.51-3.54) |
| 6.70 | 3.53 (3.51-3.55) |
| 6.80 | 3.54 (3.52-3.56) |
| 6.90 | 3.55 (3.53-3.57) |
| 7.00 | 3.56 (3.54-3.58) |
| OD (1:8100) | **Estimated titer (95% CI)** |
| 0.40 | 3.57 (3.54-3.61) |
| 0.50 | 3.64 (3.61-3.67) |
| 0.60 | 3.70 (3.67-3.73) |
| 0.70 | 3.75 (3.72-3.78) |
| 0.80 | 3.80 (3.77-3.83) |
| 0.90 | 3.85 (3.82-3.88) |
| 1.00 | 3.89 (3.87-3.92) |
| 1.10 | 3.94 (3.91-3.97) |
| 1.20 | 3.98 (3.95-4.01) |
| 1.30 | 4.02 (3.99-4.05) |
| 1.40 | 4.06 (4.03-4.09) |
| 1.50 | 4.10 (4.06-4.13) |
| 1.60 | 4.13 (4.10-4.17) |
| 1.70 | 4.17 (4.14-4.21) |
| 1.80 | 4.21 (4.17-4.25) |
| 1.90 | 4.25 (4.21-4.29) |
| 2.00 | 4.28 (4.24-4.32) |
| 2.10 | 4.32 (4.28-4.36) |
| 2.20 | 4.36 (4.31-4.40) |
| 2.30 | 4.39 (4.35-4.44) |
| 2.40 | 4.43 (4.38-4.48) |
| 2.50 | 4.47 (4.42-4.52) |
| 2.60 | 4.51 (4.45-4.56) |
| 2.70 | 4.55 (4.49-4.60) |
| 2.80 | 4.59 (4.52-4.65) |
| 2.90 | 4.62 (4.56-4.69) |
| 3.00 | 4.67 (4.60-4.73) |
| 3.10 | 4.71 (4.64-4.78) |
| 3.20 | 4.75 (4.67-4.82) |
| 3.30 | 4.79 (4.71-4.87) |
| 3.40 | 4.84 (4.75-4.92) |
| 3.50 | 4.88 (4.79-4.97) |
| 3.60 | 4.93 (4.84-5.02) |
| 3.70 | 4.98 (4.88-5.07) |
| 3.80 | 5.03 (4.93-5.13) |
| 3.90 | 5.08 (4.97-5.18) |
| 4.00 | 5.13 (5.02-5.24) |
| 4.10 | 5.19 (5.07-5.30) |
| 4.20 | 5.25 (5.12-5.37) |
| 4.30 | 5.31 (5.18-5.43) |
| 4.40 | 5.37 (5.24-5.50) |
| 4.50 | 5.44 (5.30-5.58) |
| 4.60 | 5.51 (5.36-5.66) |
| 4.70 | 5.59 (5.43-5.74) |
| 4.80 | 5.67 (5.50-5.84) |
| 4.90 | 5.76 (5.58-5.93) |
| 5.00 | 5.85 (5.67-6.04) |
| 5.10 | 5.96 (5.76-6.16) |
| 5.20 | 6.07 (5.86-6.28) |
| 5.30 | 6.20 (5.97-6.43) |
| 5.40 | 6.35 (6.10-6.59) |
| 5.50 | 6.51 (6.25-6.78) |
| 5.60 | 6.70 (6.41-6.99) |
| 5.70 | 6.94 (6.62-7.26) |
| 5.80 | 7.23 (6.87-7.59) |
| 5.90 | 7.61 (7.19-8.02) |
| 6.00 | 8.16 (7.66-8.65) |
| 6.10 | 9.11 (8.47-9.76) |
| 6.20 | 12.35 (11.14-13.56) |
| 6.21 | 14.54 (12.90-16.18) |
